# Tunable *p–p* Interactions Along Linear Chains Control Metavalent Bonding in Chalcogenide Heterostructures

**DOI:** 10.1002/advs.76916

**Published:** 2026-08-31

**Authors:** Sakshi Verma, Kanishka Biswas, Umesh V. Waghmare, C. N. R. Rao

**Affiliations:** ^1^ Theoretical Sciences Unit JNCASR Bangalore INDIA; ^2^ New Chemistry Unit JNCASR Bangalore INDIA; ^3^ School of Advanced Materials (SAMat) and Sheikh Saqr Laboratory Jawaharlal Nehru Centre for Advanced Scientific Research Bangalore INDIA

**Keywords:** chain structure, metavalent bonding, phase change materials, *p–p* hopping, thermoelectrics

## Abstract

We present an analysis of heterostructures of alternating layers of rocksalt structures of metavalently bonded (MVB) group‐IV (*MX*) and ionic chalcogenides (*IX*) and show that bonding heterogeneity across their semiconductor‐insulator interface arises from the tunable *p*–*p* interactions. Using Born dynamical charge ($Z^{*}$) as the key atomistic descriptor of MVB, we demonstrate that metavalent bonds parallel to the interfacial plane are unaffected by proximal ionic bonds as they constitute periodic (‐*X*‐*M*‐)*_n_* chains. In contrast, the bonds perpendicular to the interface (*‐X‐M‐X‐I‐X‐*) are spectacularly affected: characteristic $Z^{*}$ of the MVB evolve to the bulk behavior within about a nm of the interface. With comparative analysis with *I* = Sr, Cd, and Sn, we establish that interatomic *p*–*p* orbital interactions along linear atomic chains with 5 contiguous (‐*X*‐*M‐*) pairs govern the emergence, persistence, and suppression of MVB. The discontinuity at the interface has long‐range effects on the out‐of‐plane component of $Z^{*}$ of ions, which are explained using a simple model of electrostatic screening. Energies of *p* orbitals of anions and cations, and their interactions along (‐*X*‐*M*‐)*_n_* chains are shown here to be essential to MVB, which result in the tunability of thermoelectric properties of interfacial hetero‐structures of chalcogenides.

## Graphical abstract

Substitution of Sr at the Pb site within an atomic plane of PbTe perturbs the *p*–*p* hopping network. Since Sr does not support the *p*–*p* interaction key to metavalent bonding along (‐Pb‐Te‐) chains, long‐range electronic charge transfer in response to displacement of Pb is stalked at the Sr site.

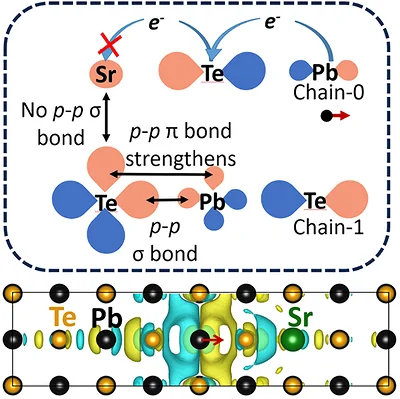

## 1 Introduction

Chemical bonding is fundamental to understanding the structure and properties of materials, such as transport, optical, and mechanical properties. Traditional types of bonding, such as covalent, ionic, metallic, and van der Waals bonding, have provided a robust framework for understanding material behavior. Interestingly, materials like PbTe [1], GeTe [2], AgSbTe_2_ [3], and Bi_2_Te_3_ [4] exhibit bonding characteristics that defy this conventional classification [5, 6, 7, 8, 9, 10, 11]. Metavalent bonding (MVB) [5, 6, 12, 13, 14], a relatively new concept, has emerged to describe the unusual combination of properties of these materials. MVB is observed in systems whose (a) electrons are neither localized nor highly delocalized, and (b) properties can't be expected from any of the traditional bonding types. Such MVB materials typically exhibit high optical dielectric constants and electrical conductivity, electronic structure with well‐dispersed bands and a narrow gap, strong coupling of phonons with electric field and anharmonicity enabling applications in thermoelectrics, infrared (IR) optics, and phase‐change memory devices [6, 10, 15, 16, 17, 18, 19, 20].

PbTe is a widely studied solid with excellent thermoelectric performance [21, 22]. MVB in PbTe is relevant to its low thermal conductivity, high Seebeck coefficient, and high electrical conductivity, the parameters that are critical to thermoelectric performance and efficiency. SrTe, on the other hand, having a rocksalt structure, has potential applications in optoelectronics and photovoltaics due to its wider bandgap and bonding of ionic character. With such bonding characters of PbTe and SrTe, their composites such as heterostructures and superlattices offer a platform of systems with tunable thermoelectric and optical properties [22, 23, 24].

The characterization of MVB involves at least five descriptors, three of which are bulk properties and the rest two are local atomistic attributes. While bulk descriptors reflect system‐averaged effects of bonding on properties, the local descriptors provide atom‐ or site‐specific insights. Bulk descriptors include electrical conductivity, the Grüneisen parameters of phonon modes (*γ*), and optical dielectric constants (*ϵ* ^∞^). The local descriptors include Born effective charge ($Z^{*}$) of an ion and effective coordination number of a site. While systematic studies of the relevance of structure through effective coordination number to MVB have been insightful [25, 26], the roles of local chemistry and orbital interactions remain unclear.

The rocksalt structure, consisting of two interpenetrating face‐centered cubic (FCC) cationic and anionic sublattices, is a prototypical structure that (i) supports each atom with octahedral coordination and (ii) becomes a simple cubic structure when anions and cations are replaced by atoms of the same species. Group‐IV chalcogenides (eg., PbTe, GeTe) in the rocksalt structure are characterized by electron‐deficient bonds, which support electron delocalization, giving “metavalent” character to the bonding. For a fixed coordination number, deviation of $Z^{*}$ of an ion from its nominal charge is a useful primary descriptor of MVB. By stacking rocksalt PbTe and SrTe into superlattices, artificial structures composed of their alternating layers, it is possible to design systems that retain the simplicity of the parent rocksalt phase while enabling the exploration of the role of local chemistry in MVB. PbTe:SrTe superlattices and Pb_1‐_ *_x_* Sr*_x_* Te contain planar interfaces across which evolution of MVB can be systematically studied. Chemical substitution at interfacial sites generates a model system in which dependence of MVB within the interface and its anisotropy can be analyzed.

Here, we explore how MVB evolves in real space from ionic bonding across an interface between ionic and MVB rocksalt crystals. We present our work in two parts. In the first part, we investigate superlattices to understand the nature of bonding at the interface. In the second part, we explore how chemical substitution influences bonding. We address specific questions such as: How do ionicity and orbitals control MVB? What is the spatial range of MVB interactions, and which structural features are key to understanding MVB and its multi‐centricity, if any?

## 2 Computational Methods

Our first‐principles calculations are within density functional theory (DFT) as implemented in the Quantum ESPRESSO (QE) package [27, 28]. Interactions between ionic cores and valence electrons were modeled using the projector augmented‐wave (PAW) method [29], and a generalized gradient approximation (GGA) is used for exchange‐correlation energy [30] with the PBE functional [31]. Potentials of Mg, Cd, O, Pb, Sr, Sn, and Te were employed with valence electron configurations of 2s^2^3s^2^2*p* ^6^, 5s^2^5*p* ^0^ *^.^* ^5^4*d* ^9^ *^.^* ^5^, 2s^2^2*p* ^4^, 5*d* ^10^6s^2^6*p* ^2^, 4*p* ^6^4s^2^5s^2^, 5s^2^5*p* ^2^4*d* ^10^, and 5s^2^5*p* ^4^, respectively. We represented electronic wave functions and charge density using a plane‐wave basis, with energy cutoffs of 48 Ry and 288 Ry for bulk PbTe and SrTe. For bulk MgO and CdO, we used higher energy cutoffs of 60 and 640 Ry, respectively. The Brillouin zone integrations were sampled with a 12 × 12 × 12 mesh of *k*‐points in the calculation of bulk structures (8 atoms per cell), and the *k*‐mesh was scaled appropriately for supercell calculations. Total energy per unit cell was converged within 10^−8^ Ry by solving the Kohn‐Sham equations iteratively to achieve a self‐consistent solution. The bulk structures were fully optimized until the residual forces on atoms were below 10^−3^ Ry/Bohr. The lattice constants (*a*), $Z^{*}$, and *ϵ* ^∞^ of bulk structures of PbTe, SrTe, MgO, and CdO are listed in Table 1. The weighted average of lattice parameters of the optimized bulk structures was used in the construction of superlattices and was kept fixed.

**TABLE 1:** Lattice constant *a*, Born effective charge $Z^{*}$, and optical dielectric constant *ϵ* ^∞^ of bulk structures. This reveals that ions of PbTe exhibit anomalous $Z^{*}$ and *ϵ* ^∞^.

Bulk	*a* (Å)	$Z^{*}$	*ϵ* ^∞^
PbTe	6.53	5.77	27
SrTe	6.71	2.42	7
MgO	4.25	1.99	3
CdO	4.77	2.46	8

Lattice dynamical properties were calculated using density functional perturbation theory (DFPT) as implemented in QE [32]. The spin‐orbit coupling (SOC) does affect the electronic structure with shifts in energy of frontier bands. While the SOC alters |$Z^{*}$(Pb)| by ∼16%, its anisotropy, which is key to the analysis here, is relatively robust (see Supporting Information). As the configurations here contain a large number of atoms, we do not include SOC in the calculations of $Z^{*}$.

## 3 Electrostatic Model of a Superlattice

**FIGURE 1:**
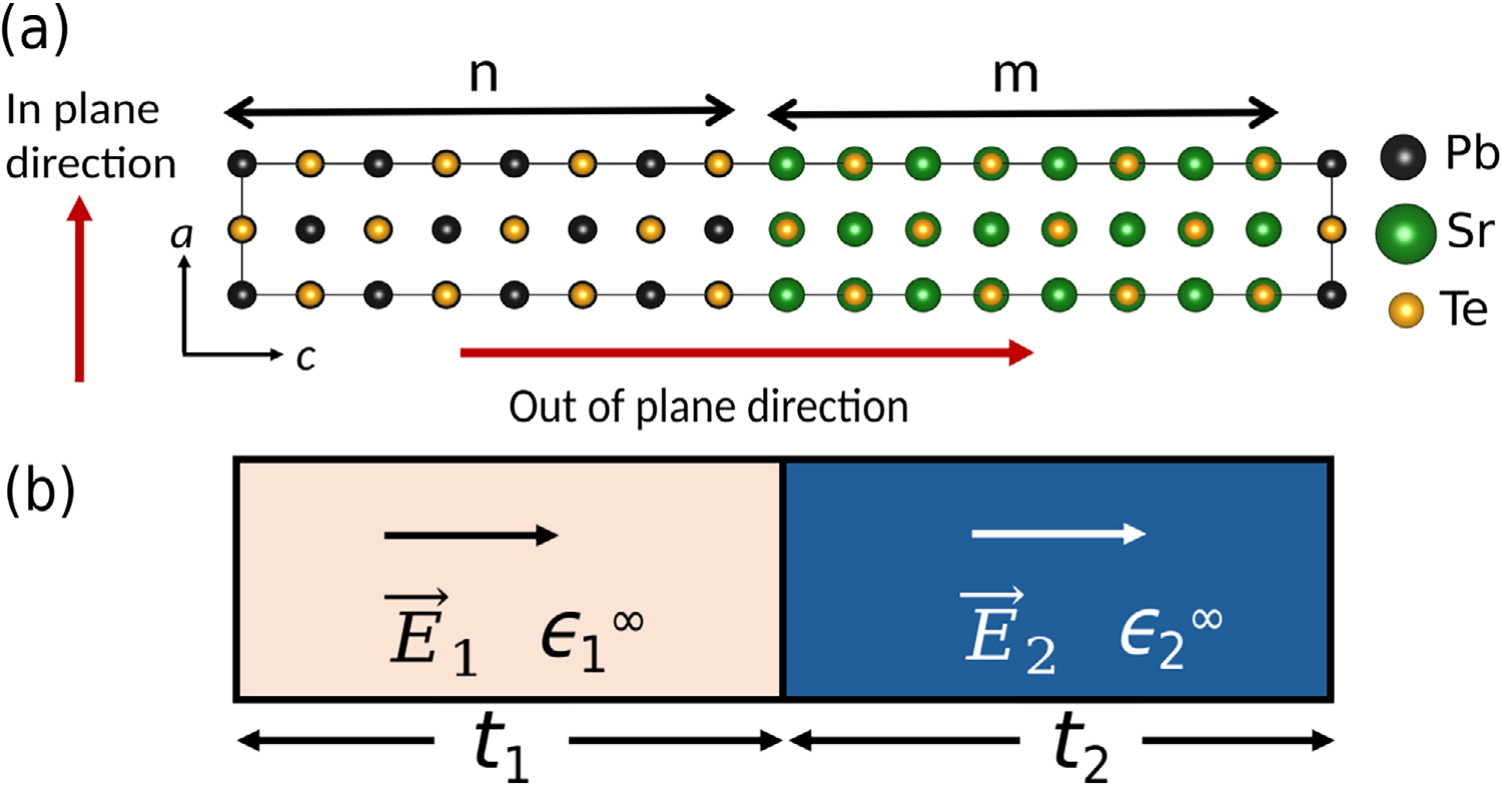
Side view (a) of (PbTe)*_n_*:(SrTe)*_m_* heterostructure or superlattice with *n* = *m* = 8. The in‐plane direction is marked by periodic (‐Pb‐Te‐) and (‐Sr‐Te‐) chains, while the out‐of‐plane direction is marked by two finite chains joining at the interface (‐Sr‐Te‐Pb‐Te‐). The model (b) used in the electrostatic analysis of the heterostructure consisting of alternating layers of thickness *t_l_* (layer‐*l*) with dielectric constant $\varepsilon_{l}^{\infty }$ and $\vec{E_{l}}$ being electric field (*l* = 1, 2).

To understand the trends in $Z^{*}$ and *ϵ* ^∞^ of superlattices, we use a simple model based on electrostatics. Using the continuity condition for the normal component of the electric displacement vector across an interface,

(1) $$\varepsilon_{1}^{\infty }E_{1}=\varepsilon_{2}^{\infty }E_{2}=\varepsilon^{\infty }E$$

where $\varepsilon_{i}^{\infty }$ is the optical (*ω* → ∞) dielectric constant of the bulk form of layer‐*i* (Figure 1b). *E_i_* is the electric field inside layer‐*i*, and *E* is the magnitude of the applied electric field. The total thickness (along the *c*‐direction) of the superlattice is *t*. *t_i_* and $f_{i}\left(=t_{i}/t\right)$ are the thickness and fractional thickness of layer‐*i*, respectively.

(2) $$E\left(t_{1}+t_{2}\right)=E_{1}t_{1}+E_{2}t_{2}$$

Substituting for *E*_1_ and *E*_2_ from Equation (1) and cancelling out *E* on both sides,

(3) $$t_{1}+t_{2}=\varepsilon^{\infty }\left[\left(\frac{t_{1}}{\varepsilon_{1}^{\infty }}\right)+\left(\frac{t_{2}}{\varepsilon_{2}^{\infty }}\right)\right]$$

Upon further simplification, the out‐of‐plane component of dielectric constant *ϵ* ^∞^of the superlattice ($\varepsilon_{\mathrm{sl}}^{\infty }$) is:

(4) $$\varepsilon_{\mathrm{sl}}^{\infty }=\frac{\varepsilon_{1}^{\infty }\varepsilon_{2}^{\infty }}{\varepsilon_{1}^{\infty }f_{2}+\varepsilon_{2}^{\infty }f_{1}}\;\;$$

which is what would be the estimate from the DFPT calculation of the superlattice. $Z^{*}$, which is defined as the change in atomic forces with change in electric field:

(5) $$Z_{i,\alpha \beta }^{*}=\frac{\partial F_{i,\alpha }}{\partial E_{\beta }}\;$$

where *i* is the atomic index, *α* is the direction of force, and *β* is the direction of the electric field. Calculated $Z^{*}$ of the *i* ^th^ atom of layer‐1 in the superlattice along the perpendicular direction (denoted by the subscript *sl*) is expressed as:

(6) $$Z_{1,i,sl}^{*}=\frac{\partial F_{i}}{\partial E}\;$$

or

(7) $$Z_{1,i,\mathrm{sl}}^{*}=\frac{\partial F_{i}}{\partial E_{1}}\frac{\partial E_{1}}{\partial E}=Z_{1,i}^{*}\frac{\varepsilon_{\mathrm{sl}}^{\infty }}{\varepsilon_{1}^{\infty }}$$

Here, $Z_{1,i}^{*}$ and $\varepsilon_{1}^{\infty }$ are the Born charges and optical dielectric constant of layer‐1 in its bulk form, respectively. Using Equation (4) in Equation (7), we get,

(8) $$Z_{1,i,\mathrm{sl}}^{*}=Z_{1,i}^{*}\;\frac{\varepsilon_{2}^{\infty }}{\varepsilon_{1}^{\infty }f_{2}+\varepsilon_{2}^{\infty }f_{1}}$$

The correctness of the model is validated considering two cases, where an analytic, exact answer is known. First, where both the layers are identical. Here, $\varepsilon_{1}^{\infty }=\varepsilon_{2}^{\infty }=\varepsilon^{\infty }$. $f_{1}=f$ and $f_{2}=1-f$. Using these values in Equation (8), we get $Z_{1,i,sl}^{*}=Z_{1,i}^{*}$ and $\varepsilon_{\mathrm{sl}}^{\infty }=\varepsilon^{\infty }.$ In the second case, we have a slab and vacuum. Here, $\varepsilon_{1}^{\infty }=\varepsilon^{\infty }$ and $\varepsilon_{2}^{\infty }=1.\;\;\;f_{1}=\;1\;-\delta$ and $f_{2}\;=\delta$. Using these in Equation (8), in the limit *δ* → 0, we get the bulk limit $Z_{1,i,sl}^{*}=Z_{1,i}^{*}$ and $\varepsilon_{\mathrm{sl}}^{\infty }=\varepsilon^{\infty }$. Both these results are exact and physically sensible, thus establishing the reliability of this model.

## 4 Results and Discussion

### 4.1 Sharp Interface: Spatial Evolution From Ionic Bonding to Metavalent Bonding

We first present a systematic analysis of MVB in periodic (PbTe)*_n_*:(SrTe)*_m_* superlattices (Figure 1a) consisting of alternating [001] oriented layers of PbTe and SrTe with *n* and *m* number of [001] atomic planes respectively, focusing here on *n* = *m* = 8. Due to periodicity in all three directions, there are two PbTe:SrTe interfaces in this structure. Due to a larger difference between the electronegativity of cations and anions, the SrTe layer exhibits ionic bonding, while PbTe is metavalently bonded. As shown in Table 1, Z^*^ and *ϵ* ^∞^ (the descriptors of MVB) of bulk PbTe are anomalous, confirming its MVB, in contrast to SrTe, which exhibits nominal $Z^{*}$ and dielectric responses as expected from ionic bonding (slight deviation of $Z^{*}$ from ideal nominal charge suggests a mix with weak covalency). To uncover the contrast between MVB and ionic bonding, we consider a purely ionic superlattice (CdO)_8_:(MgO)_8_ for comparison. The ionic character of MgO and CdO is evident in their Z^*^ and *ϵ* ^∞^ (see Table 1).

While *ϵ* ^∞^ is a bulk property, $Z^{*}$ is defined for each atom in the periodic unit cell. In a superlattice, we examine $Z^{*}$ of various ions as a function of atomic distance with respect to the interface, given in terms of plane index or count. $Z^{*}$ of an ion in the cubic rocksalt lattice is a 3 × 3 diagonal matrix with identical values of *xx*, *yy*, and *zz* components. In a superlattice, (a) the symmetry is lowered, and $Z^{*}$ of an ion takes distinct values of *zz* and (*xx* = *yy*) components (called perpendicular or out‐of‐plane and parallel or in‐plane components, respectively), and (b) their values should reach bulk‐like behavior far from the interface. Monitoring $Z^{*}$ of ions in the vicinity of the interface allows us to determine how the MVB or ionic bonding are influenced by the local chemical environment. As each atom in such a superlattice has the coordination number 6 (as in the bulk), it is an ideal platform for analysis of chemical tuning of MVB.

**FIGURE 2:**
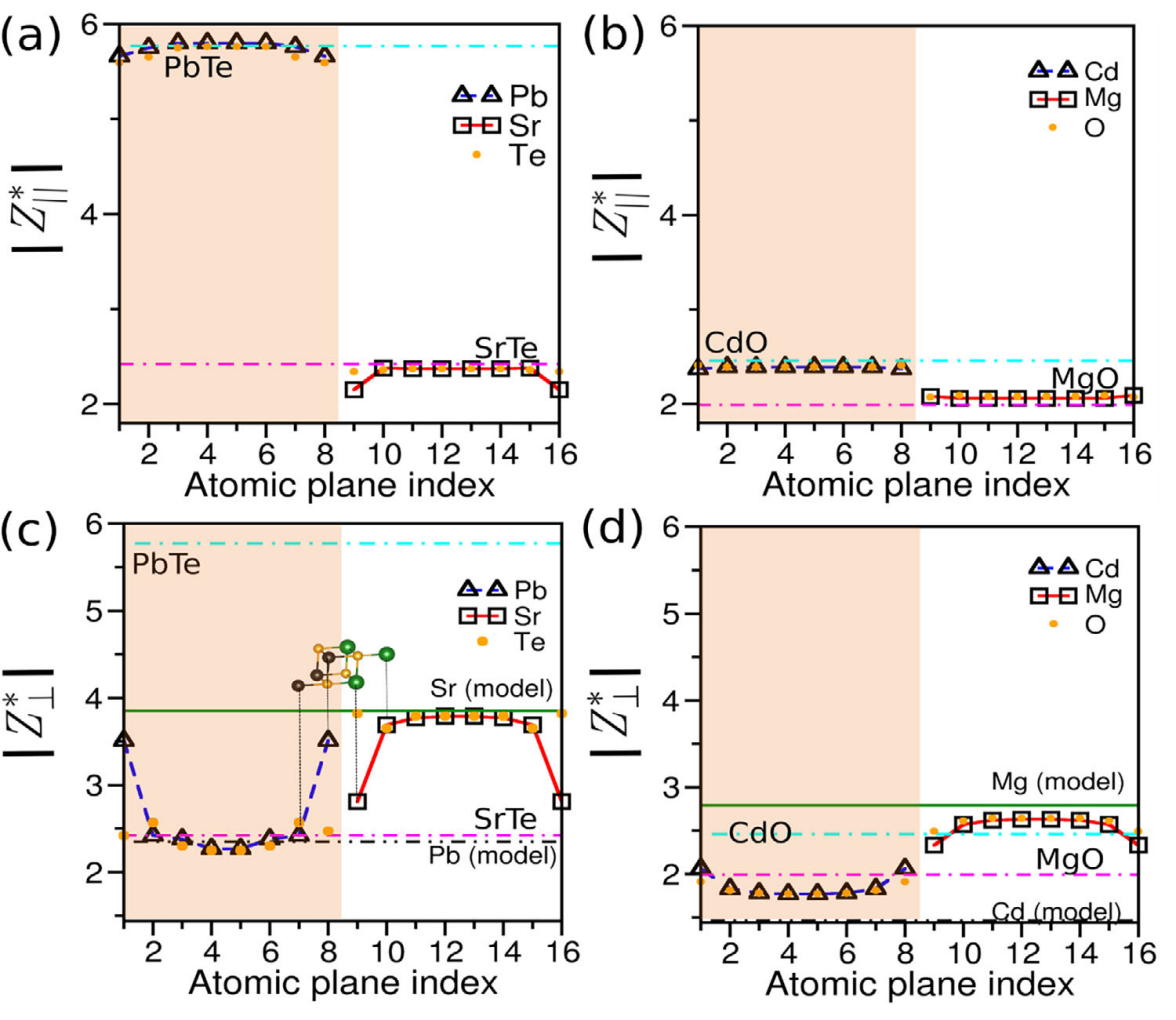
Inhomogeneity in Born charges, the descriptor of MVB, in 8:8 heterostructures. Modulus of in‐plane Born charges (denoted by $|Z_{\left|\right|}^{*}|$) of ions (Pb, Sr, and Te) in (PbTe)_8_:(SrTe)_8_ (a) and ions (Cd, Mg, and O) in (CdO)_8_:(MgO)_8_ (b) vs. the atomic plane index (1–16). Modulus of out‐of‐plane Born charges (denoted by $|Z_{⊥}^{*}|$) of ions in (PbTe)_8_:(SrTe)_8_ {with an inset showing the interfacial structure: Pb7 (Pb8) is connected to Sr9 (Sr10) via a bridging Te atom} (c) and ions in (CdO)_8_:(MgO)_8_ (d) vs. atomic plane index. Orange shaded regions represent the PbTe (CdO) layer, and white regions indicate the SrTe (MgO) layer in the superlattice.

We find that $Z^{*}$ of ions in a superlattice exhibits a remarkable anisotropy between the in‐plane (*x* and *y*) and out‐of‐plane (*z*) directions. As shown in Figure 2a,b, the in‐plane values of $Z^{*}$ of ions in the superlattice (denoted by $Z_{\left|\right|}^{*}$) closely resemble their bulk counterparts with a sharp (step function) change at the interface. While the in‐plane components of $Z^{*}$ of cations in the immediate vicinity of the interface of PbTe:SrTe (Figure 2a) exhibit weak deviation, such interfacial reduction in $Z^{*}$ is not observed at the interface in CdO:MgO (Figure 2b). We note here that the character of a rocksalt structure (continuous (‐*M*‐Te‐) chains) is preserved in the in‐plane direction.

We find spectacular anomalies in the out‐of‐plane bonds (the direction along which the continuity of (‐Pb‐Te‐) chains is disrupted by the presence of Sr) and components of $Z^{*}$ (see Figure 2c,d). The interface has a rather long‐range effect on the out‐of‐plane components of Z^*^ (denoted by $Z_{⊥}^{*})$. There is a suppression (enhancement) of $Z^{*}$ of ions in the centre of PbTe and CdO (SrTe and MgO) layers in their respective superlattices in comparison with their bulk counterparts. This is somewhat counter‐intuitive as bulk‐like behavior is expected to recover far from the interface, but can be explained with a simple model. For (PbTe)_8_:(SrTe)_8_, the fractional thicknesses, $f_{1}$ and $f_{2}$, are 0.5. Using the electrostatic model, we express $Z^{*}$ as: $Z_{1,i,\mathrm{sl}}^{*}=Z_{1,i}^{*}\frac{2\varepsilon_{2}^{\infty }}{\varepsilon_{1}^{\infty }+\varepsilon_{2}^{\infty }}$ and $Z_{2,j,\mathrm{sl}}^{*}=Z_{2,j}^{*}\;\frac{2\varepsilon_{1}^{\infty }}{\varepsilon_{1}^{\infty }+\varepsilon_{2}^{\infty }}.$ Taking the ratio of these two, $\frac{Z_{1,i,\mathrm{sl}}^{*}}{Z_{2,j,\mathrm{sl}}^{*}}=\frac{Z_{1,i}^{*}}{Z_{2,j}^{*}}\frac{\varepsilon_{2}^{\infty }}{\varepsilon_{1}^{\infty }}$ which explains the reverse changes in the magnitudes of $Z^{*}$ of ions in the centre of the respective layers (Figure 2c). Such spatial reversal of $Z^{*}$ suggests a corresponding reversal of infrared (IR) oscillator strengths. This implies that a crystal with MVB increases the IR absorption in ionic solids interfacing with it. The mechanism of this effect lies in the screening of electrostatic fields present within layers of the superlattice. $Z^{*}$ is defined as

(9) $$Z^{*}=\frac{\delta F_{Pb}\;}{\delta E_{applied}\;}\;$$

and the applied electric field *E*_applied_ is reduced by the depolarization field *E_d_* originating from the surface charges accumulating at the interface (*E* = *E*_applied_ − *E_d_*). The electric field within the PbTe layer is given by:

(10) $$E_{\mathrm{PbTe}}=E_{\mathrm{SrTe}}.\frac{\varepsilon^{\mathrm{SrTe}}}{\varepsilon^{\mathrm{PbTe}}},$$

which implies that the field in the PbTe layer is suppressed by approximately a factor of 4. Therefore, we find a suppression in $Z^{*}$ of ions in the centre of the PbTe layer. Thus, $Z^{*}$ as an atomistic descriptor is quite sensitive to the electrostatic boundary conditions.

While the out‐of‐plane components of $Z^{*}$ exhibit a smooth step‐like transition between their bulk behavior across the interface of ionic CdO and MgO (Figure 2d), they evolve non‐monotonously across the interface between MVB PbTe and ionic SrTe (Figure 2c). This can be understood using the chain structure of ions at the interface (see inset of Figure 2c) and also monitoring anionic $Z^{*}$(Te). There are two distinct chains terminating as (Pb‐Te‐Sr) motif at the interface, and the monotonicity is indeed maintained along a given chain. Specifically, Pb atoms in the seventh and eighth planes (Pb7 and Pb8) are connected to Sr atoms in the ninth and tenth planes (Sr9 and Sr10), respectively, via a Te atom. Ions in these two distinct chains (Pb7‐Te‐Sr9 and Pb8‐Te‐Sr10) have different $Z^{*}$ because of the different chemical coordinations of the bridging Te ion. In the former chain, Te is coordinated to 5 Pb and 1 Sr, which makes it chemically more similar to the Te of bulk PbTe, while in the latter chain, Te is bonded to 5 Sr and 1 Pb, making it chemically closer to the Te of bulk SrTe.

We examine the in‐plane and out‐of‐plane *p*‐orbital contributions to layer‐resolved density of states (*DOS*) in (PbTe)_8_:(SrTe)_8_ (Figure S1), and find that *p_z_* orbitals exhibit qualitatively different behavior than *p_x_* or *p_y_* orbitals. The *p_x_* orbital contribution to *DOS* remains nearly the same throughout all the atomic planes of a given layer. However, there are noticeable changes in the *p_z_* contribution to *DOS* at the interface as compared to the centre of the respective layers. In the conduction band (CB), Te‐*p_z_* orbitals of the interfacial atomic plane on the SrTe side contribute more significantly to *DOS* than Sr‐*p_z_*. This indicates that some orbital character of the valence band (VB) shifts into CB, as Te‐*p* is primarily associated with VB in bulk SrTe. In the CB, atoms in the interfacial plane on the PbTe side have two prominent peaks corresponding to Te‐*p_z_* orbitals (at 2 and 3 eV respectively), whereas in the centre of the PbTe layer, there is only one prominent peak corresponding to Te‐*p_z_* (at nearly 3 eV). These differences observed in the *p_z_* contribution to *DOS* at the interface indicate that out‐of‐plane direction is significantly influenced by the introduction of Sr, owing to disruption of continuity of (‐Pb‐Te‐) chains of interacting *p_z_*‐orbitals.

### 4.2 Anomalies in Born Charges With Ionic Substitution

**FIGURE 3:**
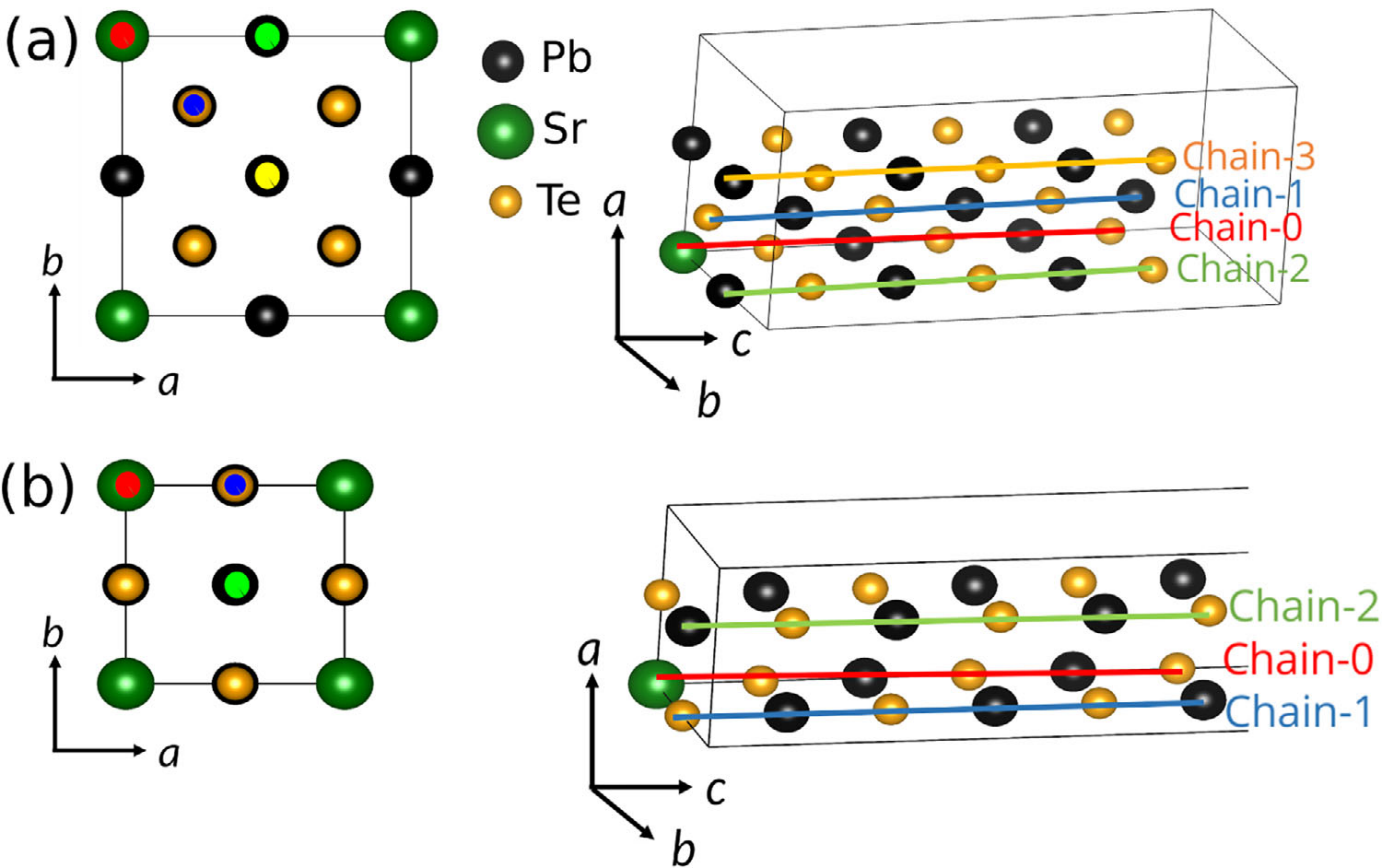
Top and perspective views of (Pb_1−_ *_x_* Sr*_x_* Te)_1_:(PbTe)*_n_* with *x* = 0.25 (a) and 0.5 (showing the interfacial region in the perspective view) (b), respectively. Different ionic chains are color‐coded. In the top view, the chains are marked by colored circles, and in the perspective view, the chains are marked by lines of respective colors.

In the *n*:*m* superlattices, an entire layer of SrTe was introduced, which resulted in suppression of $Z_{⊥}^{*}$ of ions in the PbTe layer. We now investigate the effect of dilute substitution of *A* for Pb in PbTe (where *A* = Sn, Cd, and Sr), which effectively introduces a single atomic plane rather than a complete layer. Learning from the importance of chain‐like structure, we first define the chain, focusing on the ones perpendicular to the interface in (Pb_1−_ *_x_A_x_* Te)_1_:(PbTe)*_n_* heterostructures (Figure 3) with *n* = 5, 7, 9, and 11. We introduce a nomenclature where the chain containing the substituent atom is designated as chain‐0 (marked in red). The nearest neighbor chain parallel to chain‐0 is labelled as chain‐1 (marked in blue), the second nearest chain as chain‐2 (marked in green), and the third nearest chain as chain‐3 (marked in yellow).

**FIGURE 4:**
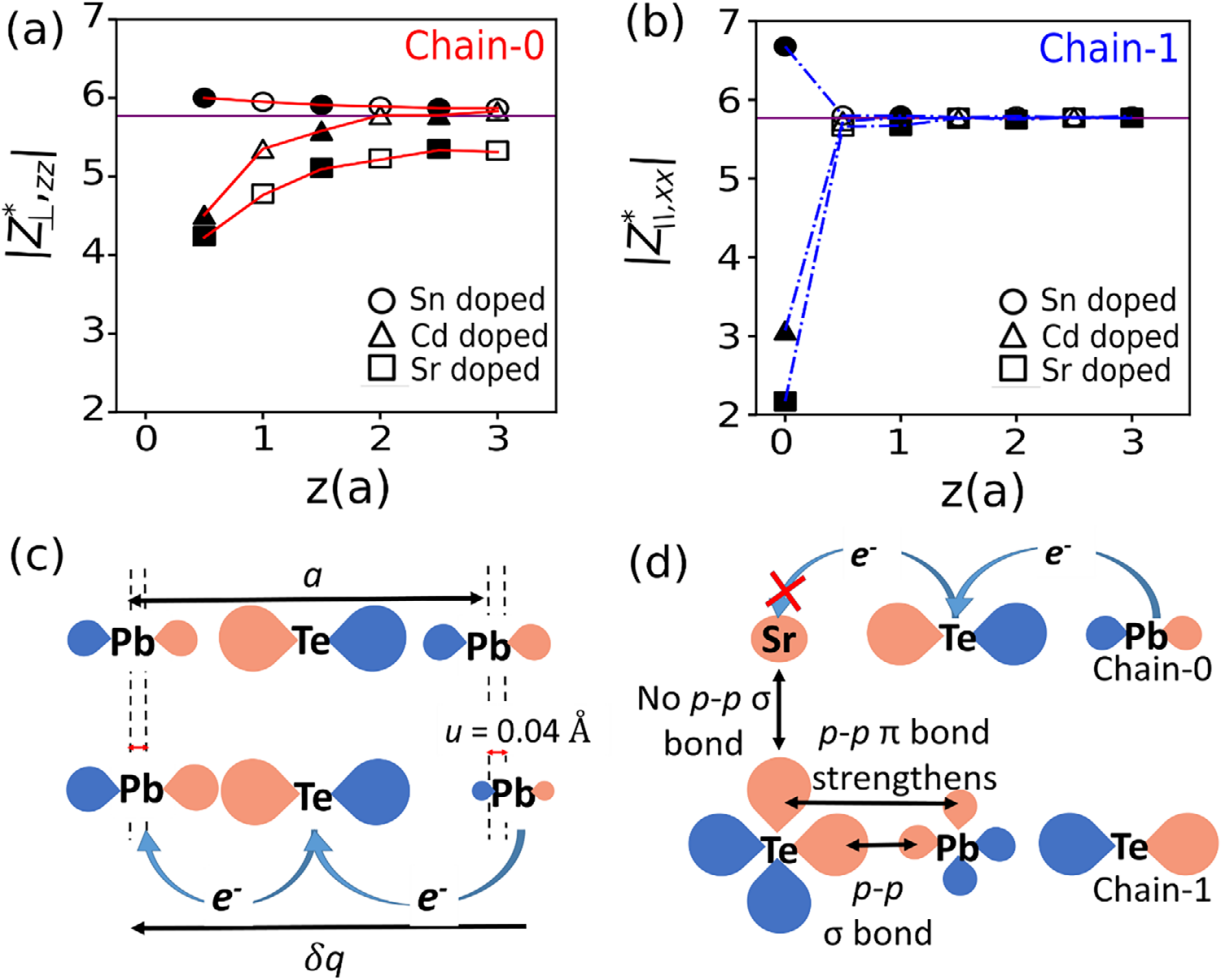
Position (*z*) dependent absolute values of out‐of‐plane (a) and in‐plane (b) Born charges of Pb (hollow symbols) and Te (filled symbols) in Pb_23_ *A*Te_24_ (*a* × *a* × 6*a*) configuration with 50% planar substitution for Pb by *A*, with *A* = Sn, Cd, and Sr. $Z_{⊥}^{*}$ of ions in chain‐0 highlight the importance of *p*–*p* hopping. $Z_{\left|\right|}^{*}$ of ions in chain‐1 approach the bulk value of 5.77, indicated by the violet line, except for the first Te atom, which belongs to the (‐*A*‐Te‐)*_n_* chain. Z^*^ of ions along other chains are covered in Figure S2. Mechanism of *p*–*p* orbital interactions in bulk PbTe (c) and in chain‐0 and chain‐1 of Sr‐doped PbTe (d). When Sr is substituted for a Pb atom, electron hopping is disabled by the lack of *p* orbitals in Sr in frontier states. This accounts for the observed drop in $Z^{*}$ in chain‐0. With no *p*–*p σ* bond between Sr and Te (from chain‐1), Te‐*p* orbitals form a stronger *π* bond with neighboring Pb‐*p* along chain‐1. This results in highly anomalous $Z^{*}$ of neighboring ions in the adjacent chains (See Figure 5a).

$Z^{*}$ in the out‐of‐plane (*zz*) direction of ions along chain‐0 (Figure 4a) exhibits similar trends (drop in $Z^{*}$ of ions) in $Z^{*}$ profiles in Cd‐ and Sr‐doped configurations, whereas the Sn‐doped configuration exhibits essentially no variation with *z*, and $Z^{*}$ of all the ions are anomalous. This confirms the role of *p*–*p* orbital interactions between anions and cations. This can be understood by considering the bonding mechanism in PbTe (Figure 4c), where small ionic displacements of the Pb sublattice induce charge transfer with electron hopping through a bridging Te ion, resulting in the formation of a net (large) dipole moment. Notably, Sn possesses valence *p* orbitals that enable such *p*–*p* interaction, thereby preserving the long‐range (across unit cell) charge transfer mechanism and maintaining the profile of anomalous $Z^{*}$ and MVB. However, as shown in Figure 4d, substitution of Pb with Sr (Cd) disrupts this process, as Sr (Cd) lacks the requisite *p* orbitals necessary for the continuity of MVB interactions, leading to a suppression of this mechanism and hence the corresponding reduction in the $Z^{*}$ of ions observed within chain‐0 of the Sr‐ and Cd‐doped configurations.

$Z^{*}$ in the in‐plane directions (*xx* and *yy*) of ions in all the chains shows saturation of $Z^{*}$ at bulk values (Figure S2). Interestingly, chain‐1, along the *xx* direction at *z* = 0, which denotes the (‐*A*‐Te‐)*_n_* chain, exhibits a Te ion with anomalous $Z^{*}$ in the first neighbor site of the Sn‐doped configuration, which is in contrast with the Cd‐ and Sr‐doped configurations (Figure 4b). We note that $Z_{\left|\right|}^{*}$ of Te in chain‐1 here is analogous to $Z_{⊥}^{*}$ of the Te ion in chain‐0 of the superlattice discussed earlier, following a similar profile. Indeed, when the continuity of *p*–*p* interactions along chains is broken in two directions, the anisotropy in $Z^{*}$ remains robust, but a bit weaker (see Supporting Information) by about 10%. This further establishes the importance of chain structure, and the contrast between $Z^{*}$(Te) of Sn and Cd/Sr substituted structures reaffirms the role of *p*–*p* interaction.

**FIGURE 5:**
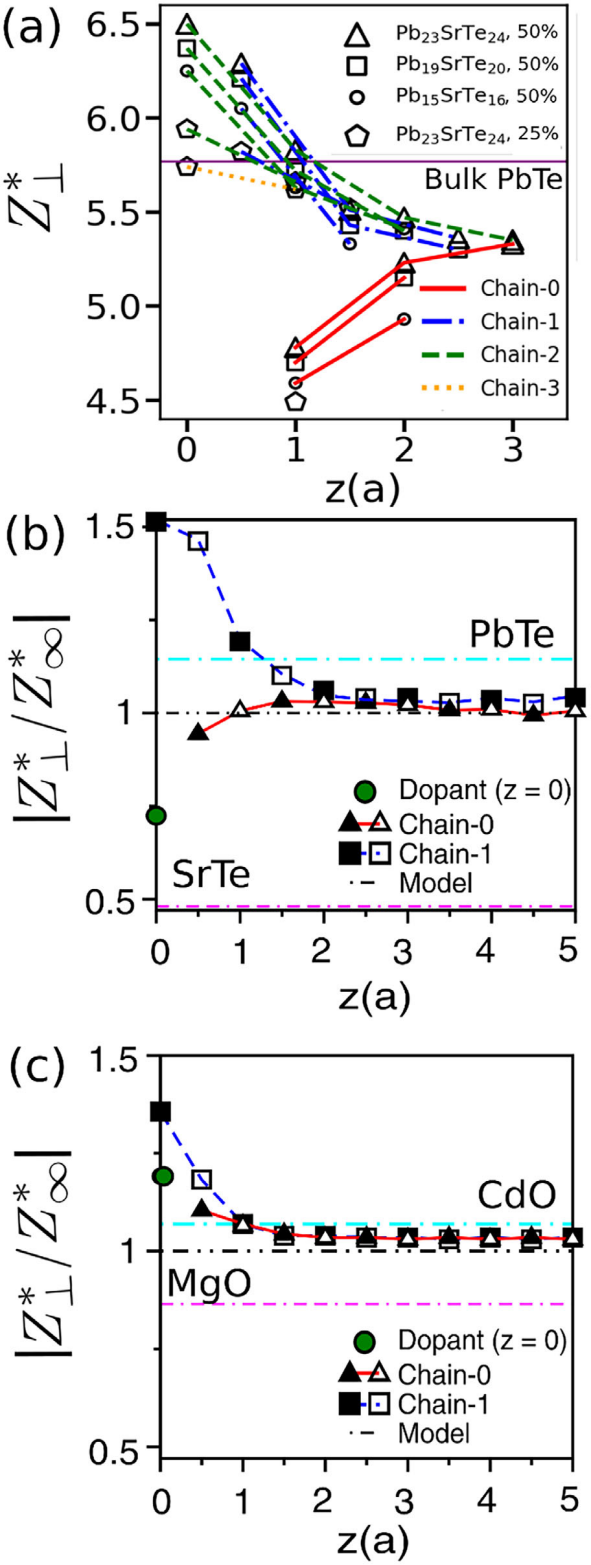
Spatial variation of $Z^{*}$ in the out‐of‐plane direction of (Pb_1−_ *_x_* Sr*_x_* Te)_1_:(PbTe)*_n_* with *x* = 0.25 and 0.5 (a) and 1 (b). Variation of $Z_{⊥}^{*}$ along chain‐0 is fundamentally different from that along chains 1–3. In terms of the influence of Sr substitution on $Z_{⊥}^{*}$, chain‐1 *>* chain‐2 *>* chain‐3. Variation of $Z_{⊥}^{*}$ in (Cd_1−_ *_x_* Mg*_x_* O)_1_:(CdO)*_n_* with *x* = 1 is provided (c) for comparison with (b). $Z^{*}$ of ions are normalized wrt the model estimates ($|Z_{⊥}^{*}/Z_{\infty }^{*}|$) of Pb in (b) and Cd in (c). Here, filled symbols denote $Z^{*}$ of anions (Te and O) and hollow symbols represent those of cations (Pb and Cd).

Having understood the effect of substitution of Pb by different cations (Sn, Cd, and Sr) within an atomic plane of PbTe, we now investigate the effect of different in‐plane doping concentrations in (Pb_1−_ *_x_* Sr*_x_* Te)_1_:(PbTe)*_n_* with *x* = 0.25, 0.50, and 1. We find that Z^*^ of ions in chain‐0 behaves radically differently from those in any other chain, marked by a drop in its $Z^{*}$ profile (as shown in Figure 5a). This is because Sr lacks *p*‐orbitals (as discussed in Figure 4d) at energies comparable to that of Te‐*p* orbitals. Second, at any value of *z*, $Z_{⊥}^{*}$(Pb) in chain‐1 deviates the most from the $Z^{*}$ of Pb in bulk PbTe, followed sequentially by chain‐2 and chain‐3. This is expected because the greater proximity to Sr leads to more drastic changes in $Z^{*}$ of ions belonging to adjacent chains.

Different chains are characterized by different coordination environments for Te ions near the interface. For 25% planar substitution, the interfacial Te ion in chain‐0 at *z* = 0.5*a* is bonded to 5 Pb and 1 Sr (out‐of‐plane), whereas the interfacial Te ion in chain‐1 is bonded to 5 Pb and 1 Sr (in‐plane) at *z* = 0. In contrast, interfacial Te ions in chains 2 and 3 (at *z* = 0.5*a*) are bonded to 6 Pb ions. For 50% planar substitution, interfacial Te (at *z* = 0.5*a*) of chain‐0 has 5 Pb and 1 Sr (out‐of‐plane) neighbors, whereas that in chain‐1 (at *z* = 0) and chain‐2 (at *z* = 0.5*a*) has 4 Pb and 2 Sr (in‐plane) neighbors and 6 Pb neighbors, respectively. The chains adjacent to chain‐0 exhibit a significant anomaly at the PbTe:SrTe interface, despite maintaining a continuous (‐Pb‐Te‐) linkage. Thus, the influence of the substituted Sr ion is not confined to chain‐0. As shown in Figure 4d, the missing *p*–*p σ* bond between Sr (Cd) and Te (from chain‐1) causes the Te‐*p* orbitals to engage in stronger *π* interactions with *p* orbitals of neighboring ions along chain‐1. This increased *π* bonding interaction leads to even more anomalous $Z^{*}$ of the first few ions in the adjacent chains. Furthermore, we note saturation of $Z^{*}$ of Pb with respect to *z* (at ≈ 3*a*) to a value much lower than the bulk value. This too can be understood using the simple model presented earlier. In the case of 50% Sr planar substitution in the structure comprising a total of 12 atomic planes of PbTe (Figure 3b), we employ a weighted averaging approach to estimate $Z^{*}$ of ions and find that the estimated value (5.49) is quite comparable to the converged value (5.33).

$$Z_{\mathrm{Pb},\mathrm{zz}}^{*}=Z^{*}\frac{0.5\varepsilon_{\mathrm{SrTe}}^{\infty }+0.5\varepsilon_{\mathrm{PbTe}}^{\infty }}{\varepsilon_{\mathrm{PbTe}}^{\infty }\frac{1}{12}+\varepsilon_{\mathrm{SrTe}}^{\infty }\frac{11}{12}}=5.49$$

In the (Pb_1−_ *_x_* Sr*_x_* Te)_1_:(PbTe)_19_ and (Cd_1−_ *_x_* Mg*_x_* O)_1_:(CdO)_19_ superlattices with *x* = 1 (Figure 5b,c), only chain‐0 and chain‐1 are present. Distinction between these two chains can be made by analysing the coordination environment of anions (Te or O) at the interface. For the (Pb_1−_ *_x_* Sr*_x_* Te)_1_:(PbTe)_19_ system, the Te ion at *z* = 0.5*a* in chain‐0 has 5 Pb and 1 Sr (out‐of‐plane) neighbors, whereas that in chain‐1 at *z* = 0 has 2 Pb and 4 Sr (in‐plane) neighbors. As shown in Figure 5b,c,  $Z^{*}$ of ions in the chain‐0 closely follows $Z_{\infty }^{*}$ (where $Z_{\infty }^{*}$ denotes the model‐estimated $Z^{*}$ of Pb and Cd along the out‐of‐plane direction in their respective superlattices using Equation 8). However, chain‐1 exhibits a strong and opposite spatial dependence (due to *p*–*p π* bonding interactions as explained earlier and shown in Figure 4d). In the (Pb_1−_ *_x_* Sr*_x_* Te)_1_:(PbTe)_19_ superlattice, the saturation of $Z^{*}$ of ions of both chains with respect to *z* occurs within 2.5 unit cells. In contrast, it converges within 1.5 unit cells in the (Cd_1−_ *_x_* Mg*_x_* O)_1_:(CdO)_19_ superlattice. Although the analysis is based purely on electrostatics, saturation of $Z^{*}$ at different *z* values highlights the long‐range effects of electrostatic screening. Furthermore, the deviation of $Z^{*}$ of ions in chain‐1 from their bulk values is more pronounced in the (Pb_1−_ *_x_* Sr*_x_* Te)_1_:(PbTe)_19_ superlattice ($\Delta \frac{Z_{⊥}^{*}}{Z_{\infty }^{*}}=0.28$), as compared to (Cd_1−_ *_x_* Mg*_x_* O)_1_:(CdO)_19_.

Spatial inhomogeneities in $Z^{*}$ result in enhanced scattering of charge and heat carriers and hence reduced carrier mobility, ultimately impacting the thermoelectric performance. Moreover, saturation of $Z^{*}$ within 2.5–3 unit cells indicates the involvement of 5–6 contiguous bonds along linear chains. Thus, the mechanism of MVB is not quite explained with the concept of 3‐center, 4‐electron (3*c*–4*e*) bonding in Ref. [33], as the 3*c* picture involves only 2 bonds. Thus, the spatial dependence of $Z^{*}$ in the PbTe:SrTe superlattices reported here points to *p*–*p* orbital interactions extending to 4–5 *M*‐*X* bonds along a linear chain, consistent with the picture in Ref. [25]. Indeed, the rocksalt structure is very special in the sense that such ionic chains run along all three directions!

## 5 Conclusions

We demonstrated that the linear chain‐like structure of alternating anions and cations and their *p*–*p* orbital interactions are fundamental to understanding MVB. On substitution of Sr for Pb, MVB in PbTe gets suppressed due to the lack of Sr‐*p* orbitals in the frontier states. In particular, the spatial variation of $Z^{*}$ along different atomic chains reveals that the disruption in *p*–*p* orbital hopping dominates the suppression of anomalous $Z^{*}$ of ions in the chains of MVB chalcogenides. In contrast, for adjacent chains, stronger *p*–*p π* interactions enhance the anomaly in $Z^{*}$. In addition to highlighting the contrast between MVB and ionic bonding, we demonstrate that MVB involves electronic interactions extending up to 2–3 unit cells, which helps in retaining good carrier mobility. Our work establishes a framework for understanding and engineering MVB in complex materials, particularly involving interfaces and nano‐precipitates that are key to high‐performance thermoelectrics, opening up applications in solar‐thermo‐electric energy conversion and substrate‐enhanced infrared absorption (see Supporting Information).

## Author Contributions

**Sakshi Verma**: conceptualization, investigation, writing – original draft, formal analysis, methodology, writing – review and editing. **Kanishka Biswas**: validation, Writing – review and editing. **Umesh V. Waghmare**: conceptualization, investigation, funding acquisition, resources, writing – review and editing, writing – original draft, methodology, validation, formal analysis, supervision. **C. N. R. Rao**: validation, writing – review and editing.

## Conflicts of Interest

The authors declare no conflicts of interest.

## Supporting information




**Supporting File**: advs76916‐sup‐0001‐SuppMat.docx.

## Data Availability

The data that support the findings of this study are available from the corresponding author upon reasonable request.
