# Supplementary material for: Tunable p–p Interactions Along Linear Chains Control Metavalent Bonding in Chalcogenide Heterostructures
Source: Adv Sci (Weinh). 2026 Aug 31;13(59):e76916. doi: 10.1002/advs.76916 (PMC13529253; doi:10.1002/advs.76916)
Supplement: Supplementary file 1 [file ADVS-13-e76916-s001.docx]

**Supplementary Information for Tunable *p*-*p* Interactions along Linear Chains Control Metavalent Bonding in Chalcogenide Heterostructures**

*Sakshi Verma, Kanishka Biswas, Umesh V. Waghmare* and C. N. R. Rao**

Umesh V. Waghmare, C. N. R. Rao

School of Advanced Materials (SAMat) and Sheikh Saqr Laboratory, Jawaharlal Nehru Centre for Advanced Scientific Research, Jakkur P. O., Bangalore 560064, INDIA

E-mail: waghmare@jncasr.ac.in, [cnrrao@jncasr.ac.in](mailto:cnrrao@jncasr.ac.in)

Sakshi Verma, Umesh V. Waghmare

Theoretical Sciences Unit, JNCASR, Jakkur P. O., Bangalore 560064, INDIA

Kanishka Biswas, C. N. R. Rao

New Chemistry Unit, JNCASR, Jakkur P.O., Bangalore 560064 INDIA

Keywords: Metavalent bonding, Chain structure, p-p hopping, Thermoelectrics, Phase Change Materials

1. **Application**

Motivated by the anomalous $Z^{*}$ responses to field in the out-of-plane direction, we delve into its practical implications. We consider a periodic unit of the superlattice in capacitive geometry with $L_{P}$ being the thickness of PbTe layer and $L_{S}$ being the thickness of SrTe layer (Figure S3a).

Using continuity of electric displacement fields, thermal current across interface assuming $\sigma_{S}\approx0$ such that electric current vanishes, we get for the capacitive device (see Figure S3a)

$$T_{S}=\frac{L_{S}E}{k_{S}}\left( \frac{k_{P}}{\epsilon_{P}S_{P}}-\frac{\sigma_{P}S_{P}T_{P}}{2\epsilon_{P}} \right)=\frac{L_{S}E}{k_{S}}\left( \frac{k_{P}}{\epsilon_{P}S_{P}}+\frac{\sigma_{P}L_{P}E}{2\epsilon_{P}^{2}} \right)$$

where $E$ is the applied static electric field, $\epsilon_{P}$ ($k_{P}$) and $\epsilon_{S} (k_{S})$ are the dielectric constants (thermal conductivities) of PbTe and SrTe respectively, $S_{P}$($\sigma_{P}$) is the Seebeck coefficient (electrical conductivity) of PbTe and $T_{P}$ is the temperature at the end surface of PbTe.

We substitute $T_{P}/2$ for the average *T* in PbTe layer, assuming linear variation in *T*.

Thus, the temperature difference generated capacitively across the PbTe:SrTe device using $k_{S}\gg k_{P}$ is

$$\Delta T\approx\left( \frac{L_{P}}{\epsilon_{P}S_{P}} \right)\left[ 1+\frac{E_{P}L_{S}\left( \sigma_{P}S_{P} \right)}{2k_{S}} \right]E$$

where the factor $\left( \frac{L_{P}}{\epsilon_{P}S_{P}} \right)$ is the bare contribution of PbTe and the enhancement factor $\frac{E_{P}L_{S}\left( \sigma_{P}S_{P} \right)}{2k_{S}}$ is formally similar to $zT$ ($zT=\frac{S^{2}\sigma T}{k}$ is similar to $\frac{\left( S_{P}\sigma_{P} \right)(E_{P}L_{S})}{k_{S}}$). This means that the temperature difference across PbTe:SrTe device is enhanced relative to pristine PbTe when SrTe is used as a thermal contact. Importantly, the enhancement factor is tunable with electric field. There is minimal heat loss as $J_{e}=0$, effectively enhancing $zT$. We now estimate an electric field for which the enhancement factor is ≈ 2.

$$E_{P}L_{S}=\frac{k_{S}}{\sigma_{P}S_{P}}$$

Thus, $E_{P}=0.037 V/Å$ for the 8:8 superlattice (see subsection 1.1 for detailed analysis) which can be achieved experimentally.

Interface of SrTe with PbTe enhances $Z^{*}$ of atoms in SrTe (along the out-of-plane direction), which are the coupling between polar phonons and IR field. We thus expect enhanced absorption selectively in SrTe layer [$I\left( \omega\right)\propto{{(Z}^{*})}^{2}$]. We term this as the substrate (here PbTe) enhanced IR absorption. We propose a device based on this phenomenon (Figure S3b), wherein, p-type PbTe and n-type PbTe are connected to each other via a metal strip and are interfaced with SrTe. When IR radiation is incident on the device along the in-plane direction such that the corresponding electric field is along the out-of-plane direction, SrTe layer gets selectively heated up due to electrostatically enhanced $Z^{*}$, which leads to generation of thermoelectric current in the circuit. Thus, the proposed device can be used to convert low energy component of solar energy into heat energy and subsequently to electrical energy. This is complementary to photovoltaic solar cells which mostly rely on the high energy, ultraviolet (UV) component of the solar spectrum.

**1.1 Expression for temperature difference generated capacitively (as shown in Figure S3a)**

Continuity of electric displacement fields gives:

$$\frac{E}{\epsilon_{P}}L_{P}+\frac{E}{\epsilon_{S}}L_{S}=E. \left( L_{P}+L_{S} \right)=V$$

where $E$ is the applied static electric field, *V* is the voltage, $\epsilon_{P}$ and $\epsilon_{S}$ are the dielectric constants of PbTe and SrTe respectively.

Since electrical conductivity of SrTe is negligible, $\sigma_{S}\approx0$, we assume that the electrical current, $J_{e}$ through the device vanishes. However, the thermal current, $J_{Q}$ can be non-zero in the steady state.

The condition of vanishing electric current $J_{e}=\sigma\left[ E-S\nabla T \right]$ gives

$$J_{e}= \sigma_{P}\left[ E_{P}-\frac{S_{P}\left( T_{I}-T_{P} \right)}{L_{P}} \right]=0$$

where $S_{P}$ is the Seebeck coefficient of PbTe, $T_{I}$ and $T_{P}$ are the temperatures at the interface and at the end surface of PbTe respectively. Substituting for $E_{P}(=E/\epsilon_{P})$ we get

$$\frac{E}{\epsilon_{P}}L_{P}=S_{P}\left( T_{I}-T_{P} \right)$$

The thermal current is $J_{Q}=\pi J_{e}-k\nabla T$, where $k$ is thermal conductivity, $\pi$ is Peltier coefficient and $J_{e}=\sigma E$. For PbTe and SrTe layers, we get

$$J_{Q}= -\frac{k_{P}\left( T_{I}-T_{P} \right)}{L_{P}}+\pi_{P}\sigma_{P}E_{P}= -\frac{k_{S}\left( T_{S}-T_{I} \right)}{L_{S}}$$

For simplifying analysis, we set $T_{I}=0 K$ as the reference temperature we get from $J_{e}=0$:

$\frac{EL_{P}}{\epsilon_{P}S_{P}}= -T_{P}$	(S1)

and

$$\frac{k_{P}}{L_{P}}.T_{P}+\frac{\pi_{P}\sigma_{P}E}{\epsilon_{P}}+\frac{k_{S}T_{S}}{L_{S}}=0$$

Substituting for $T_{P}$ from Eq. S1,

$$-\frac{k_{P}}{\epsilon_{P}S_{P}}.E+\frac{\pi_{P}\sigma_{P}E}{\epsilon_{P}}+\frac{k_{S}T_{S}}{L_{S}}=0$$

Using $\pi=ST$, and continuity of thermal current $J_{Q}$ we get

$$T_{S}=\frac{L_{S}E}{k_{S}}\left( \frac{k_{P}}{\epsilon_{P}S_{P}}-\frac{\sigma_{P}S_{P}T_{P}}{2\epsilon_{P}} \right)=\frac{L_{S}E}{k_{S}}\left( \frac{k_{P}}{\epsilon_{P}S_{P}}+\frac{\sigma_{P}L_{P}E}{2\epsilon_{P}^{2}} \right)$$

where we substitute $T_{P}/2$ for the average T in PbTe layer.

Thus, the temperature difference generated capacitively across the PbTe:SrTe device is

$$\Delta T=T_{S}-T_{P}=\left[ \frac{L_{S}k_{P}}{\epsilon_{P}S_{P}k_{S}}+\frac{L_{P}}{\epsilon_{P}S_{P}} \right]E+\frac{\sigma_{P}L_{S}L_{P}E^{2}}{2k_{S}\epsilon_{P}^{2}}$$

Rearranging the terms on RHS, we get using $k_{S}\gg k_{P}$

$$\Delta T=\left[ \frac{k_{P}}{\epsilon_{P}S_{P}}\left( \frac{L_{S}}{k_{S}} \right)+\frac{L_{P}}{\epsilon_{P}S_{P}} \right]E+\frac{1}{2}\left( \frac{L_{S}}{k_{S}} \right)\left( \frac{\sigma_{P}L_{P}}{\epsilon_{P}^{2}} \right)E^{2}$$

As $k_{S}\gg k_{P}$, ${L_{S}/k}_{S}\ll{L_{P}/k}_{P}$.

$$\Delta T=\left[ \frac{{(L}_{S}/k_{S})}{\epsilon_{P}S_{P}}+ \frac{{(L}_{P}/k_{P})}{\epsilon_{P}S_{P}} \right]k_{P}E+\frac{1}{2}\left( \frac{L_{S}}{k_{S}} \right)\left( \frac{\sigma_{P}L_{P}}{\epsilon_{P}^{2}} \right)E^{2}$$

$$=\left[ \frac{L_{S}}{k_{S}}+\frac{L_{P}}{k_{P}} \right]\left( \frac{k_{P}}{\epsilon_{P}S_{P}} \right)E+\frac{1}{2}\left( \frac{L_{S}}{k_{S}} \right)\left( \frac{\sigma_{P}L_{P}}{\epsilon_{P}^{2}} \right)E^{2}$$

$$\Delta T\approx\left( \frac{L_{P}}{\epsilon_{P}S_{P}} \right)\left[ 1+\frac{E_{P}L_{S}\left( \sigma_{P}S_{P} \right)}{2k_{S}} \right]E$$

Substituting the reported values of $\sigma_{P}$ (≈ 1000 $\Omega^{-1}cm^{-1}$), $S_{P}$ (≈ 200 $\mu VK^{-1}$) and $k_{S}$ (≈ 9.66 $Wm^{-1}K^{-1}$) at 300 K: ^[3,4]^

$$E_{P}L_{S}=\frac{9.66\times{10}^{4}}{1000\times200} V$$

$$=0.966 V$$

For an 8:8 superlattice, $L_{S}=25.895 Å$, we get

$$E_{P}=\frac{0.966}{25.895}V/Å$$

$E_{P}=0.0373 V/Å$.

**1.2 Efficiency of the solar-thermoelectric device (as shown in Figure S3b)**

The efficiency of such a device cannot quite readily be estimated as its figure of merit is not established yet. Our objective has been therefore not to predict a quantitative efficiency, but rather to demonstrate the underlying mechanism and identify the material parameters governing a device based on SrTe:PbTe heterostructure that promotes selective absorption of IR in the SrTe layer.

For the SrTe:PbTe solar device, we use (a) the MVB to get enhanced thermoelectric conversion efficiency ($\eta_{\mathrm{te}}$), and (b) heterostructure to generate selective heating of SrTe by IR absorption. Thus, the temperature difference between hot and cold ends (∆*T* = *T*_h_ - *T*_c_) is amplified. The generated photocurrent originates from the temperature difference established across the heterostructure due to IR absorption. Using a model discussed by Gang Chen,^[5]^ one may obtain the solar-to-electric conversion efficiency.

2. **Chain mediated nature of bonding**

We analyzed PbSr_35_Te_36_ (3*a* × 3*a* × *a*) and Pb_6_Sr_30_Te_36_ (3*a* × 3*a* × *a*) as shown in Figure S6a & b respectively, in which the -Pb-Te- chains remain continuous along the *c* direction (defined here as the “in-plane” direction in terms of uninterrupted *p*-*p* linkage), while they are interrupted by Sr substitution along both *a* and *b* directions (out-of-plane). Pb, Sr and Te are represented by black, green and orange spheres respectively. We find that in Figure S6a, $Z^{*}$ of Pb is anomalously high along *c* (5.26), close to its bulk PbTe value (5.77), whereas it decreases notably to 3.48 along *a* and *b* directions. Such directional dependence demonstrates that metavalent bonding is supported by continuous chains of group IV chalcogenides and is significantly weakened when the continuity of *p*-*p* hopping along a chain is interrupted. In Figure S6b, $Z^{*}$ of Pb ions remain anomalously large along the chain $c$ direction (5.35-5.60), approaching the bulk value (5.77) closer than that in Figure S6a. This arises from greater similarity with bulk PbTe enhancing the Pb-Te *p*-*p* interactions compared to that in Figure S6a. In contrast, the $Z^{*}$values along *a* and *b* directions decrease significantly, falling within the range of 3.37-4.27. Indeed, when the continuity of *p*-*p* interactions along chains is broken in two directions, the anisotropy in $Z^{*}$ remains robust, but a bit weaker (the anisotropy ratio $Z_{\mathrm{out}}^{*}/Z_{\mathrm{in}}^{*}$ is 0.66 and 0.72 in Figure S6a and Figure S6b respectively. These results therefore provide further support for the proposed chain-mediated nature of the bonding.

3. **Effect of SOC on** $\boldsymbol{Z}^{\boldsymbol{*}}$

To determine the impact of SOC on $Z$^*^, we simulated (PbTe)_2_:(SrTe)_2_ superlattice as shown in Figure S5a. Here, *a* and *b* are the in-plane directions and *c* is the out of plane direction. As shown in Table S1, SOC does alter the absolute values of $Z^{*}$of Pb ions by upto 16% along the in-plane direction and 4% along the out-of-plane direction. However, we find that their anisotropy remains robust even upon inclusion of SOC (in fact enhanced by 10% due to the SOC): Pb atoms consistently exhibit larger in-plane than out-of-plane Born charges ($Z_{\mathrm{in}}^{*}>Z_{\mathrm{out}}^{*}$), while Sr atoms exhibit the opposite trend ($Z_{\mathrm{out}}^{*}>Z_{\mathrm{in}}^{*}$). The anisotropy ratio $Z_{\mathrm{out}}^{*}/Z_{\mathrm{in}}^{*}$ for Pb remains within 10% of ≈ 0.6 with and without SOC. Therefore, SOC alters the values of $Z$^*^, while their unusual anisotropy is quite robust.

**Table S1**: Comparison of Born effective charges ($Z^{*}$) along the in-plane ($Z^{*}$_in_) and out-of-plane ($Z^{*}$_out_) directions for cations in (PbTe)_2_:(SrTe)_2_ superlattice, calculated without (wosoc) and with SOC (wsoc).

| **Layer #** | **Cations** | $\boldsymbol{Z}^{\boldsymbol{*}}$**_out_** | | $\boldsymbol{Z}^{\boldsymbol{*}}$**_in_** | | $\boldsymbol{Z}^{\boldsymbol{*}}$**_out_/**$\boldsymbol{Z}^{\boldsymbol{*}}$**_in_** | |
| --- | --- | --- | --- | --- | --- | --- | --- |
|  |  | **wosoc** | **wsoc** | **wosoc** | **wsoc** | **wosoc** | **wsoc** |
| 1 | Pb1, Pb4 | 3.54 | 3.68 | 5.41 | 6.37 | 0.65 | 0.58 |
| 2 | Pb2, Pb3 | 3.54 | 3.69 | 5.41 | 6.37 | 0.65 | 0.58 |
| 3 | Sr5, Sr7 | 2.68 | 2.77 | 2.13 | 2.06 | 1.26 | 1.34 |
| 4 | Sr6, Sr8 | 2.68 | 2.74 | 2.13 | 2.06 | 1.26 | 1.33 |

**Figure S1**: In-plane (*p_x_*) (a) and out-of-plane (*p_z_*) (b) orbital contributions to *pDOS* of (PbTe)_8_:(SrTe)_8_. The *pDOS* is shown on a range of 0 to 0.1 states/eV. The *p_x_* contribution to the *DOS* remains consistent across both the layers and the interface, while the *p_z_* contribution exhibits significant variation at the interface compared to the centre of the layers. The introduction of Sr disrupts the continuity of the (-Pb–Te-) chains and *p_z_* orbitals, strongly affecting the out-of-plane direction.

**Figure S2**: Modulus of in-plane (a-e) and out-of-plane (f-g) Born charges of Pb (hollow symbols) and Te (filled symbols) in Pb_23_*A*Te_24_ (*a* × *a* × 6*a*) configuration, with *A* = Sn, Cd and Sr as a function of spatial coordinate *z*. In-plane Born charges approach the bulk value of 5.77, indicated by the violet line. In the out-of-plane Born charges along chain-1 and chain-2, Sn- and Cd-doped configurations display similar variation of $Z^{*}$ along *z*, despite Cd lacking valence *p* orbitals. This similarity is likely due to their filled *d*^10^ electron shells and comparable atomic numbers.

**Figure S3**: Capacitive device (a) of (PbTe)_8_:(SrTe)_8_. The thickness of PbTe and SrTe layers is $L_{P}$ and $L_{S}$ respectively. The temperature at the end surfaces of PbTe and SrTe layers is $T_{P}$ and $T_{S}$ respectively, and at the interface is $T_{I}.$ The capacitive device is connected to a battery whose electric field generates the temperature difference and heat current $J_{Q}$. Due to substrate enhanced $Z^{*}$ of ions in SrTe (out-of-plane), IR radiation selectively heats up SrTe. Thus, SrTe can be used as an ideal thermal contact with PbTe, and convert solar energy to electrical energy with a two-legged device (b).

**Figure S4:** Redistribution of charge density induced by displacing a Pb ion by 0.04 Å along the *c*-direction (indicated by an arrow) in (a) PbTe and (b) Pb_15_SrTe_16_ ($a\times a\times4a$ supercell). Pb, Sr and Te are represented by black, green and orange spheres respectively. The displacement of a single Pb ion in the former (a) produces a charge redistribution that decays with distance but extends throughout the supercell. This long-range response highlights the multicentric nature of metavalent bonding (MVB). In the latter (b), such long range electronic charge transfer is stalked at Sr site. Isosurface of the charge density difference is plotted at 2 $\times$10^-5^ e Å⁻³. Yellow denotes charge accumulation and cyan denotes charge depletion.

**Figure S5:** *pDOS* of (a) (PbTe)_2_:(SrTe)_2_ (b) without SOC and (c) with SOC. Pb, Sr and Te are represented by black, green and orange spheres respectively. The primary effect of SOC is in shifting in the energy positions of electronic bands. The same orbitals govern the frontier states. Therefore, the SOC does not alter the electronic interactions qualitatively.

**Figure S6:** Chain mediated nature of bonding is further analyzed in (a) PbSr_35_Te_36_ (3*a* × 3*a* × *a*) and (b) Pb_6_Sr_30_Te_36_ (3*a* × 3*a* × *a*), in which the -Pb-Te- chains remain continuous along the *c* direction (defined here as the in-plane direction due to the uninterrupted *p*-*p* linkage), while they are interrupted by Sr substitution along *a* and *b* directions (out-of-plane). Pb, Sr and Te are represented by black, green and orange spheres respectively.

**References**

[1] P. Giannozzi et al., *J. Phys. Condens. Matter*, 2009, **21**, 395502.

[2] P. Giannozzi et al., *J. Phys. Condens. Matter*, 2017, **29**, 465901.

[3] S. C. Rakesh Roshan et al., [*ACS Applied Energy Materials*](https://pubs.acs.org/journal/aaemcq?ref=breadcrumb), 2021, **5**, 882-896.

[4] C. J. Vineis et al., *Phys. Rev. B*, 2008, **77**, 235202.

[5] G. Chen, *J. Appl. Phys.,* 2011, **109**, 104908.
